# Innovation or acquisition? Emissions mitigation strategies and the role of renewable technologies

**DOI:** 10.1371/journal.pone.0316020

**Published:** 2024-12-31

**Authors:** Zahra Moqadassi, Iman Miremadi, Hossein Khajehpour

**Affiliations:** 1 Graduate School of Management and Economics, Sharif University of Technology, Tehran, Iran; 2 Energy Engineering Department, Sharif University of Technology, Tehran, Iran; Sichuan University, CHINA

## Abstract

One of the actions taken to mitigate the climate change is research, development and demonstration (RD&D) investments in renewable energy (RE) technology. In addition to domestic RD&D spending, the import of foreign technologies, as a main channel of technology transfer, is another option to obtain higher share of renewable energies in order to achieve climate objectives. In this study, a panel dataset of 28 OECD member countries from 2011 to 2020 is analyzed, using the OLS, fixed-effects, and two-step system GMM methods, to assess the impacts of public spending on renewable energy RD&D (RERD) and the import of renewable energy technologies on the energy-related CO_2_ emissions. To fully capture the significant regional differences, the 28 countries are re-divided into four regions in accordance with their renewable energy RD&D investment level and total CO_2_ emissions. This study uniquely investigates the impact of RERD and importation–as an alternative channel for obtaining renewable energy technologies–on energy-related CO_2_ emissions in OECD countries, while also analyzing regional differences to inform targeted local policies. The findings reveal that CO_2_ emission is significantly and negatively affected by renewable energy imports, for the full panel as well as for Low-RERD and Low-Emission regions. Furthermore, only in High-RERD and High-Emission regions can renewable energy RD&D decrease CO_2_ emissions. Accordingly, some policy implications are recommended concerning investments in renewable energy RD&D and renewables import.

## 1. Introduction

Global warming, which is the main indicator of climate change, has become one of the most concerned environmental issues over the last decades. Carbon dioxide (CO_2_) is considered to be the biggest contributor to global warming and environmental sustainability reduction [1]. Therefore, mitigating CO_2_ emissions has become a priority worldwide. However, current environmental policies are not yet sufficiently effective to limit emissions to a sustainable level [2].

The Paris Agreement requires all Parties to take actions to response to climate change by accelerating innovation and technology development [3]. Moreover, the Glasgow climate pact, agreed in 26th UN Climate Change Conference of the Parties (COP26), highlights the need to reduce greenhouse gases emissions. Changing the energy production method away from fossil fuel based systems is a priority of emission reduction strategies [4]. Technological innovation can be considered as the major driver of change [5]. In this regard, one of the actions taken to mitigate the climate change is innovation in renewable energy technologies.

The share of the renewable resources in power generation in the last decade has necessitated the increased R&D investment required for resource conservation [6]. Therefore, one of the main drivers of innovation in the energy sector is research, development and demonstration (RD&D [According to IEA, “Energy RD&D covers research, development and demonstration related to the production, storage, transportation, distribution and rational use of all forms of energy” (IEA, 2024).]) [7].

In this regard, previous studies investigated the role of renewable energy RD&D on CO_2_ emissions. However, different strands of literature found contradicting results (i.e., the literature that found a positive [8], negative [9–13], or neutral [14, 15] effects of renewable energy RD&D on CO_2_ emissions. This inconsistency shows the necessity of investigation into the specific conditions under which the renewable energy R&D may be effective in reducing CO_2_ emissions.

The introduction of new technologies may occur both through domestic R&D efforts and through international technology transfer. In the latter model, the technological gap is bridged through imitation and importation of foreign technology, rather than R&D and innovation [16]. As OECD [17] stated that policies may be suitable and effective in only specific contexts, it is essential to examine the environmental effectiveness of various climate policy options to allocate resources efficiently. This study aims to address the gap in research regarding the effectiveness of different approaches–innovation and acquisition–that countries can adopt to obtain renewable energy technologies, aiming to reduce their CO_2_ emissions based on each country’s specific context.

This study empirically investigates the impacts of renewable energy RD&D as well as renewable energy import on energy-related CO_2_ emissions in 28 OECD member countries over the period 2011–2020, employing various econometric methods. OECD member countries account for 60% of world GDP, 75% of world trade, and 50% of the world’s energy consumption (U.S. Department of State). According to the literature, economic growth, energy productivity, and renewable energy development are also considered as control variables.

The paper is organized as follows. Section 2 provides a brief review of the literature. Section 3 presents the methodology and data, section 4 points out the empirical results and discussion, and sections 5 highlights the main conclusions of the work and policy implications.

## 2. Literature review

### 2.1. Renewable energy RD&D and CO_2_ emissions

In recent years, the push for decarbonizing the energy sector has driven economies to invest in the technological advancements necessary for diversifying energy sources. Numerous researchers have explored the effectiveness of these investments in reducing emissions through various approaches.

Erdogan [18] revealed that renewable energy investments reduce petroleum-derived carbon emissions in the G7 countries. In the context of Germany, Pata [19] argued that renewable energy R&D expenditures are effective in reducing CO_2_ emissions. Leng Wong [20] showed that more R&D in renewable energy reduces fossil fuel consumption. They found that countries with and without oil reserves can effectively reduce their reliance on fossil fuel through the promotion of R&D in renewable energy. In this regard, Ahmed [14] also argued that the public investments in renewable energy RD&D is essential to develop the market for green technologies to replace fossil fuels and undoubtedly influences future climate outcomes.

By developing a model to explain the dynamics among innovation, manufacturing, and market, Zheng and Kammen [21] argued that both the deployment incentive policies and public R&D spending play important roles in stimulating technology innovation in the solar photovoltaic industry. Similarly, Lin and Zhu [22] also revealed that the investment in R&D from governments and enterprises can improve the level of renewable energy technology innovation. Miremadi [23] showed that a proper budget on energy research policy affects energy technology deployment, especially for renewable energy technologies.

Government support for renewable energy R&D can help accelerate the energy transition on the supply side [24]. Lior [25] argued that R&D and implementation of renewable energy must continue intensely, with the most promising technologies currently being wind, solar photovoltaics and solar-thermal power, and to some extent biomass. And finally, by studying selected EU states and adopting the zero unitarization method, Guzowska [26] revealed that the most environmentally efficient R&D expenditures in the energy sector were on renewable energy.

However, very few researchers scrutinized the linkage between renewable energy RD&D and CO_2_ emissions and revealed different findings. Koçak and Ulucak [15], by studying 19 high-income OECD member countries using GMM and pooled OLS techniques, provided evidence that there is no significant relationship between renewable energy R&D and CO_2_ emissions. Ahmed [14] examined the impact of renewable energy RD&D on CO_2_ emissions in the US. They showed that although the renewable energy RD&D investment in the US has not gained the required level to affect CO_2_ emissions, increasing renewable energy RD&D should be on the central agenda of the US environmental policies. On the other hand, Bilgili [8], studying 13 developed countries, argued that renewable energy R&D increases CO_2_ emissions.

However, some other studies were able to reveal the negative impact of renewable energy R&D on CO_2_ emissions, by exploring different countries. Mourshed and Quddus [27] by studying 15 European countries showed that the expenditure on renewable energy RD&D as a percentage of total government energy RD&D investment reduces CO_2_ emissions. However, they could not find a significant effect of total government energy RD&D investment on CO_2_ emissions.

Using CS-ARDL approach for seven selected developed countries, Ni [11] found a negative impact of renewable energy R&D, fiscal decentralization and institutional quality on CO_2_ emissions. By studying G7 economies from 1990 to 2019, Qin [12] showed that renewable energy R&D, environmental policy, green innovation, and composite risk index help control carbon emissions. Utilizing the time series data for the US ‘s case over the period from 1990 to 2019, Shao [13] revealed that environmental-related R&D and renewable energy R&D positively contribute to carbon neutrality target achievement by reducing atmospheric CO_2_ emissions.

Also Ahmed [9] investigated the effect of public investments in R&D related to renewable energy and technological innovation on CO_2_ emissions for G7 countries, controlling for the level of trade globalization in these countries. They showed that R&D investments in renewables are critical for curbing CO_2_ emissions in the long run. Moreover, utilizing panel dataset for 27 countries from 1980 to 2020, Hailemariam [10] examined the effect of public investment in renewable energy R&D on a spectrum of greenhouse gas emissions. They concluded that R&D investment in renewable technologies significantly reduces methane, carbon monoxide, nitrogen oxide, and CO_2_ emissions.

### 2.2. International trade in renewable energy

Zhang [28] investigated the opportunities for potential cooperation in renewable energy between China and the United States. To this end, they established a mathematical model to characterize correlations among GDP, CO_2_ emissions, energy prices and the renewable energy cooperation (trade) index. They found that renewable energy cooperation can promote economic development, reduce CO_2_ emissions, improve the environment and realize green growth.

Countries that were not part of the first group of innovators have used various strategies to boost their renewable energy development, including transferring renewable energy technologies [29]. Renewable energy technology transfer does not necessarily mean importing the most advanced technologies from highly innovative countries, but countries may import more suitable or cost-effective technologies from their peers [30].

Analyzing low, lower-middle and upper-middle income countries across the five continents, Murshed [31] showed that the development of renewable energy mainly depends on renewable trade liberalization policies. In this regard, strengthening inter-regional cooperation in renewable energy plays an important role in the development of renewable energy [32].

Increasing imported technology may complement or substitute in-house R&D [33]. Tan and Hwang [34] revealed that the relationship between imported technology and in-house R&D is complementary. However, according to Lall [35], the relationship between technology transfer and domestic technological efforts is changeable. Indeed, at some stages, the two are substitutes while at others they may be complementary.

Determining the balance between the development of domestic knowledge (i.e. public R&D) and the advantage from knowledge developed abroad is an important issue [23]. Moreover, the more open the system, the more capability to imitate advanced foreign knowledge [36]. Therefore, in order to achieve sustainable energy transition, renewable energy technologies can be acquired both through in-house R&D and through imports. For instance, the main procedure applied by Chinese firms to acquire the technology of solar PV production from abroad was to simply import most of the equipment to manufacture modules [37]. Zhang and Gallagher [38], focusing on the development of the PV industry in China, argued that innovation in clean energy technology can occur through both global and national innovation processes, and knowledge exchange along the global PV value chain. Conte and Vivarelli [39] argued that R&D expenditures are statistically significant in enhancing product innovation, while technology acquisition significantly impacts the engaging in process innovation.

Huang [40] used a panel dataset covering China’s 30 provincial regions and concluded that both domestic R&D and import’s technology spillover play a significant role in decreasing China’s carbon intensity. Also Luan [41] utilized a panel dataset representing China’s industrial sectors from 2000 to 2010 and revealed that R&D activities and technology acquisition from domestic and abroad are conducive to reducing carbon intensity.

Although extensive literature is available on the relationship between renewables and CO_2_ emissions, there are still limited studies investigating the environmental effectiveness of renewable energy RD&D. Moreover, the only way to obtain renewable energy technologies is not domestic innovation. Governments may prefer to import foreign technologies instead of invention through domestic R&D. To the best of our knowledge, there is no study available in the literature that empirically investigates the impact of renewable energy RD&D on energy-related CO_2_ emissions while considering the role of renewable energy technology transfer through import. Moreover, despite the differences in CO_2_ emissions as well as RD&D public budgets across various regions, no studies were found that examined the regional heterogeneous effects of renewable energy RD&D on CO_2_ emissions. As previously reviewed, the literature on environmental effectiveness of renewable energy RD&D is not only very limited, but also is still inconclusive, implying an ambiguous nexus between renewable energy RD&D and CO_2_ emissions. This article, therefore, aims to contribute to the nascent yet growing literature by investigating regional effects of renewable energy RD&D and renewable energy import on energy-related CO_2_ emissions through studying countries in groups with similar characteristics.

Building on the insights from the literature, this study hypothesizes that the effectiveness of domestic development of renewable energy technologies is not the same across different contexts. The hypothesis suggest that discussed channels–RD&D and import–to obtain renewable energy technologies have different effects on CO_2_ emissions, depending on the contexts of the countries.

## 3. Methodology and data

### 3.1. Variables and data

This study employs an unbalanced panel data covering annual frequency data for 28 OECD countries over the period from 2011 to 2020, selected based on the availability of data for renewable energy RD&D budget. Panel data sample includes the following countries: Australia, Austria, Belgium, Canada, Czech Republic, Denmark, Estonia, Finland, France, Germany, Hungary, Ireland, Italy, Japan, Korea, Mexico, Netherland, New Zealand, Norway, Poland, Portugal, Slovak Republic, Spain, Sweden, Switzerland, Turkey, the United Kingdom, and the United States.

Based on the literature previously discussed, the employed variables in this analysis are selected and constructed as follows:

*Per capita CO*_*2*_ *emissions from energy (denoted as CE)*. This study uses per capita energy-related CO_2_ emission as the dependent variable. Data for the CE, measured in ton, is obtained from BP.

*Public investment in renewable energy RD&D (denoted as RERD)*. The renewable energy RD&D is the main independent variable. According to IEA (2024), renewable energy sources are defined as the “fuels and energy obtained: (i) directly from solar radiation; (ii) indirectly from its effects on the biosphere and the life within it; (iii) from geothermal energy; and (iv) from gravitational forces”. Data for RERD is collected from IEA (2024). Since there are significant time-lags between R&D inputs and outputs [42], following Guzowska [26], Koçak [43], and Liu [44], we include the two-year lag term of renewable energy RD&D in the model. We measure RERD as the share of GDP for each country, then scale it by percentage. The advantage of this transformed variable is that it makes RD&D comparable among countries with different GDPs.

*Renewable energy import (denoted as REIMP)*. We include the import value of renewable energy facilities as the independent variable in the model. The data, measured in US dollars, is obtained from the UN comtrade database. We use the HS headings classified by Gosens [45] to identify import value of renewable energy technologies and products. Similar to RERD, we measure REIMP as the percentage of the share of renewable energy import value in GDP for each country.

*Per capita GDP (denoted as GDP)*. We use this control variable as Wang [46] suggests the significance of GDP regarding carbon emissions. The data on GDP (current US dollars) and population are acquired from World Bank. We rescale GDP data to constant PPP US 2020 dollars.

*Renewable energy development (denoted as REDEV)*. This variable is added to the model since by changing the share of fossil fuels in the energy mix, it could affect the carbon emissions from energy sector [47]. Data for REDEV is taken as the percentage of the share of renewable energy sources in total electricity generation, obtained from British Petroleum.

*Energy productivity (denoted as EP)*. We included energy productivity as a control variable in our econometric model, following Shao [13]. The EP data is taken as total primary energy supply (TPES) per unit of GDP and is collected from OECD (2022). Table 1 contains the descriptive statistics for the variables.

*Annual patents filed for renewable energy technologies (denoted as RPAT)*. In order to examine the robustness of our estimation, the renewable energy RD&D is replaced by *PRAT* in the model. The reason to choose this variable is that R&D expenditure and the number of patents are commonly utilized to measure the technological innovations [48]. Data for *PRAT* is obtained from International Renewable Energy Agency.

**Table 1:** Descriptive statistics of variables.

Variable	Variable description	Mean	Std. Dev.	Min.	Max.	Obs.
*Dependent variable*						
CE	Per capita CO2 emission from energy (tonnes)	8.43	3.91	2.774178	18.67028	280
*Independent variables*						
RERD	Public renewable energy technology RD&D budget (% of GDP)	0.007	0.007	0	0.07	256
REIMP	Renewable energy technology import (% of GDP)	0.14	0.11	0.0153821	0.8597964	280
*Control variables*						
GDP	GDP per capita (Constant PPP 2020 USD)	46452.16	13652.6	18858.93	95769.16	280
REDEV	Renewable energy generation (% of total electricity generation)	34.98	24.79	2.472	98.571	280
EP	GDP per total primary energy supply	13162.75	4905.71	6157.82	36644.19	280
RPAT	Annual patents filed for renewable energy technologies	985.05	2140.87	1	11381	273

One of the major problems in panel data analysis is multicollinearity, which happens when independent variables in the regression model are highly correlated to each other. Multicollinearity may cause problems in model fitting and interpretation of the results. Therefore, the correlation statistical approach is employed to detect multicollinearity among the variables. Based on the results of correlation analyses reported in Table A. 1 in S1 Appendix, the models used for analysis won’t be involved in the obvious multicollinearity problem.

### 3.2. Empirical analysis

#### 3.2.1. Pre-estimation tests

For the data having the cross-sections and time-series at the same time, it is imperative to check for the stationarity. In this regard, we employ Augmented Dickey-Fuller (ADF) unit root test presented by Dickey and Fuller [49], for checking the issue of structural breaks. The test assumes the presence of the unit root in the data as the null hypothesis. The results of the ADF test are provided in Table A. **2** in S1 Appendix. The unit root test’s empirical estimations found all the variables stationary either at level or at first difference.

Studies in empirical macroeconomics almost always involve nonstationary and trend variables. In such a case, spurious regression occurs when regressing two non-stationary variables. However, if the variables are cointegrated, then spurious regression is no longer a problem. In the case that two series are cointegrated, we can assume a long-run relationship between variables regardless of whether they are I(0) or I(1) [50]. In this regard, we employ the Kao [51] and Westerlund [52] cointegration tests. The results, shown in Table A. 3 in S1 Appendix, suggest that all the variables are cointegrated.

#### 3.2.2. Full panel estimation

In this study, panel data analysis is used to estimate the casual effect of renewable energy RD&D and renewable energy imports, and the control variables on energy-related CO_2_ emissions.

Our general model can be written as:

$$CE_{it}=f(RERD_{it}+REIMP_{it}+GDP_{it}+REDEV_{it}+EP_{it})$$ (1)

where *CE*, *RERD*, *REIMP*, *GDP*, *REDEV*, and *EP* represent the variables extensively discussed in section 3.1., for country *i* at time *t*. The natural logarithms of the variables are used to directly estimate the impacts of the explanatory variables on the percentage change of the dependent variable.

Following Ullah [53], we use a step-by-step procedure to test the applicability of the methods: (1) to perform an OLS estimation and check whether there might be an endogeneity problem of the independent variables. If the endogeneity exists, it needs to be solved; otherwise, OLS estimate is valid; (2) to perform fixed-effects model and show whether the dynamic endogeneity is captured; and (3) to tackle the endogeneity issue by applying a two-step system GMM method.

*Step 1*: *OLS analysis*. Owing to its wide usage in prior research, initially, we carry out an OLS analysis. The related results are shown in the first column of Table 2. As the amount of CO_2_ emissions may affect the explanatory variables of the model, there is a potential of endogeneity problem that can lead to biased inferences. Thus, the results of the OLS estimate are inconsistent due to the potential problem of endogeneity.
*Step 2*: *Fixed-effects estimation*. The fixed-effects estimation technique is employed which can control for the individual fixed effects. We also control for the time-specific effect. The results are reported in the second column of Table 2. Fixed-effects estimation is a static panel data model, which means the impact of the explanatory variables on CO_2_ emissions has no time lag effect [54]. However, the relationship between renewable energy RD&D and CO_2_ emissions is lagged and dynamic [11–13]. Consequently, fixed-effects panel data static models would lead to inconsistent and biased estimates in this case [54].
*Step 3*: *Two-step system GMM estimation*. Finally, in order to address the endogeneity problem issues arising from reverse causality, we apply the general method of moments (GMM) that includes the lagged dependent variable as the instrumental variable. The system GMM model, developed by Arellano and Bover [55] and Blundell and Bond [56], increases the number of instruments and provides more efficient estimates as compared to the difference GMM. Since the one-step system GMM could result in the loss of too many observations, the two-step system GMM model, recommended by Arellano and Bover [55], is adopted to analyze the driving factors of *CE*. Our two-step system GMM model is presented in the following equation:
$$\begin{array}{ll} lnCE_{it} & =\alpha +\beta_{1}lnCE_{it-1}+\beta_{2}lnRERD_{it-2}+\beta_{3}lnREIMP_{it}\\ & +\beta_{4}lnGDP_{it}+\beta_{5}lnREDEV_{it}+\beta_{6}lnEP_{it}+\varepsilon_{it} \end{array}$$ (2)
  where *CE*_*it*−1_ indicates one lag of the dependent variable, representing per capita energy-related CO_2_ emission in the previous year, and *ε*_*it*_ is the error term. The corresponding results are provided in the last column of Table 2.

**Table 2:** OLS, fixed effects, and two-step system GMM models.

Variable	OLS	FE	GMM
*lnCE*_*it*−1_			0.641***
			(0.111)
*lnRERD*_*it*−2_	-0.086***	0.001	-0.005
	(0.023)	(0.005)	(0.024)
*lnREIMP*	0.010	-0.011	-0.073***
	(0.028)	(0.009)	(0.023)
*lnGDP*	0.919***	1.222***	0.462***
	(0.073)	(0.088)	(0.140)
*lnREDEV*	-0.219***	-0.114***	-0.282***
	(0.026)	(0.022)	(0.086)
*lnEP*	-0.827***	-1.012***	-0.197
	(0.055)	(0.098)	(0.136)
_*Cons*	0.285	17.740***	-1.626*
	(0.810)	(3.931)	(0.950)
*AR*(2)			0.417
*Hansen test*			0.478
*Obs*.	209	209	196

Note: Robust standard errors in parenthesis.

***, ** & * is for 1%, 5% and 10% level of significance.

The consistency of a system GMM estimator depends on the validity of the instruments, which is examined by applying the Hansen test. Failure to reject the null hypothesis supports the selected instruments. Moreover, the test for autocorrelation of the error terms called AR(2) examine whether the error terms are serially correlated. These two tests indicate that the regression results are reliable.

#### 3.2.3. Regional heterogeneous analysis

**Fig 1:**
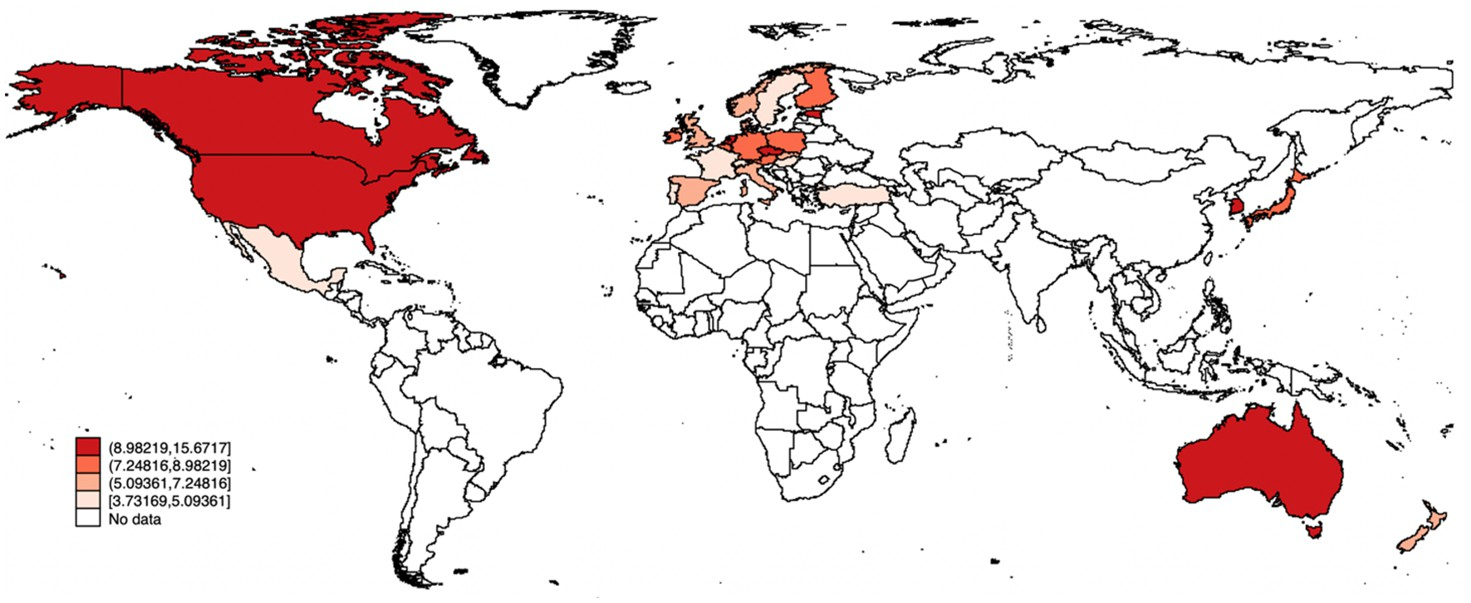
The spatial distribution of per capita total CO_2_ emissions (2015–2020 average). Significant differences exist in per capita total CO_2_ emissions across various regions in the OECD countries sample.

**Fig 2:**
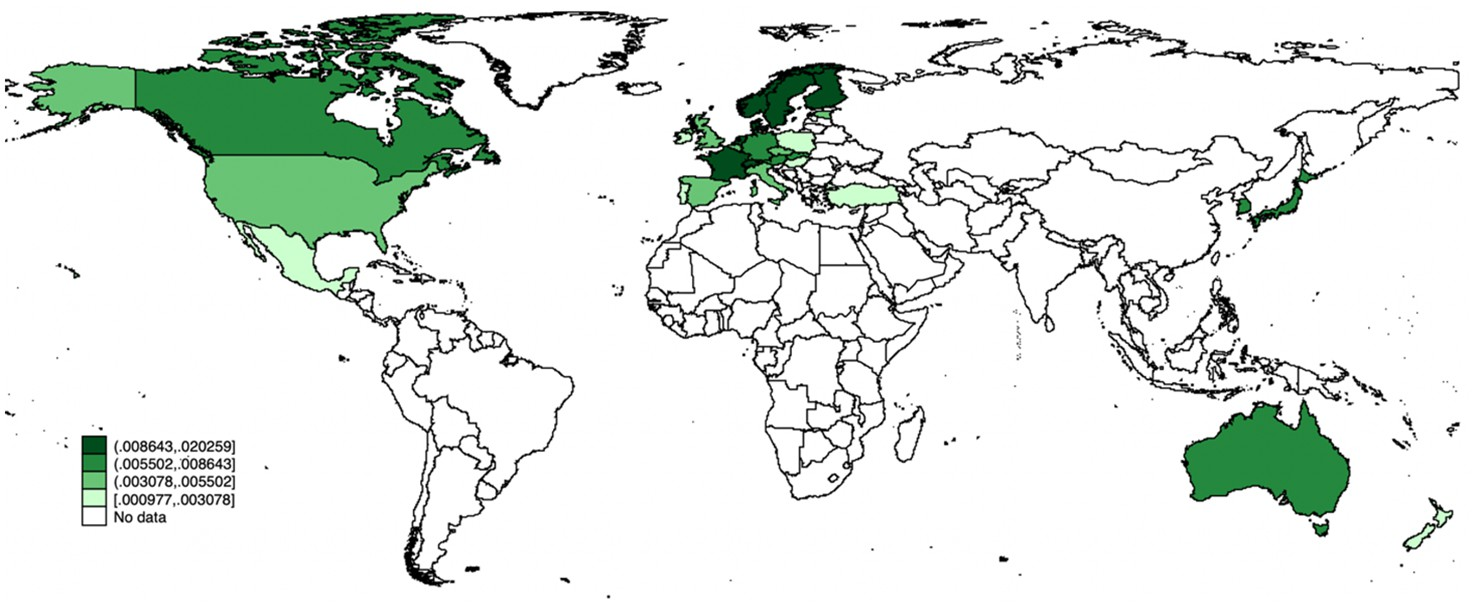
The spatial distribution of the percentage of the share of renewable energy RD&D public investments in GDP (2011–2020 average). Significant differences exist in the share of renewable energy RD&D in GDP across various regions in the OECD countries sample.

As shown in Figs 1 and 2, significant differences exist in the share of renewable energy RD&D in GDP and per capita total CO_2_ emissions across various regions in the OECD countries sample. Accordingly, this research’s critical question arises: How can renewable energy RD&D and renewable energy import affect energy-related CO_2_ emissions across various regions?

To answer this question, we re-divide the 28 countries into four regions in accordance with their *RERD* and total per capita CO_2_ emission levels by expanding the work of Jiang [57] and Zhao [58]: High-RERD region, Low-RERD region, High-Em region, and Low-Em region. The classification criteria can be divided into the following steps: (1) Calculating the average values of *RERD* from 2011 to 2020 and the average values of total per capita CO_2_ emissions from 2015 to 2019 for each country; (2) Calculating the average values of *RERD* and total per capita CO_2_ emissions for the whole panel over the mentioned time periods; and (3) Classifying the 28 countries into the four regions. The countries of each region are listed in Table A.4 in S1 Appendix and also shown in Fig 1.

We employ the two-step system GMM method to detect the different impacts of *RERD* and *REIMP* on *CE* across various regions; the results for the regions are provided in Table 3.

**Table 3:** Results of the regional analysis (two-step system GMM).

Variables	High-RERD	Low-RERD	High-Em	Low-Em
*lnRERD*_*it*−2_	-0.033**	0.040	-0.050**	-0.023
	(0.014)	(0.024)	(0.020)	(0.081)
*lnREIMP*	-0.031	-0.078**	-0.026	-0.141***
	(0.021)	(0.027)	(0.054)	(0.046)
*lnGDP*	1.158**	0.339**	1.340***	0.422*
	(0.381)	(0.159)	(0.343)	(0.205)
*lnREDEV*	-0.197***	-0.143***	-0.380***	-0.144***
	(0.054)	(0.036)	(0.079)	(0.044)
*lnEP*	-0.212*	-0.317**	-0.035	-0.103
	(0.097)	(0.133)	(0.339)	(0.203)
_*Cons*	-9.902**	0.593	-13.423**	-3.039
	(3.452)	(0.639)	(6.124)	(2.914)
*AR*(2)	0.143	0.365	0.291	0.685
*Hansen test*	0.977	0.161	0.992	0.295
*Obs*.	78	118	86	102

Note: Robust standard errors in parenthesis.

***, ** & * is for 1%, 5% and 10% level of significance.

#### 3.2.4. Robustness test

In order to assess the robustness of our estimation, we conduct a robustness test on both the whole sample and the regional samples. The two-step system GMM approach is maintained for this analysis. Following Lin and Zhu [22], instead of using *lnRERD*_*it*−2_, the annual patents filed for renewable energy technologies (*lnRPAT*_*it*−2_) are directly utilized for the robustness assessment. In the technology development literature, R&D expenditure and the number of patents are widely used to measure the technological innovations. The former is generally taken as the input of innovation, while the latter is employed as the output [48]. The results are presented in Table 5.

## 4. Results and discussion

### 4.1. Empirical results

We used the OLS model, the fixed-effects model, and the two-step system GMM model for the entire sample. The empirical results are provided in the Table 2.

In the fixed-effects model, the coefficients of the lagged term of renewable energy RD&D are not statistically significant even at the 10% significance level, while the coefficient is significantly negative in the OLS model. Furthermore, renewable energy development and energy productivity have negative impacts on carbon emission, while per capita GDP positively affects carbon emission. Additionally, no significant effect of renewable energy import on CO_2_ emissions is found.

Next, we applied the two-step system GMM model on the whole sample; the results of the estimation and the diagnostic tests are provided in the third column of Table 2. The p-value of the Hansen test is 0.478, so it demonstrates that the applied instruments are valid. Since the p-value of AR(2) is 0.417, the null hypothesis of no autocorrelation cannot be rejected. These diagnostic tests indicate that the regression results are reliable.

The GMM results could be different than those reported in the OLS and the fixed-effects columns. Using two-step system GMM, the negative effect of renewable energy import on CO_2_ emissions becomes stronger and statistically significant. Similar to the results of the fixed-effects model, renewable energy RD&D is not a significant determinant of carbon emission. We can see that the coefficient of *lnCE*_*it*−1_ is positive and statistically significant, implying that the energy-related CO_2_ emission has path-dependence and the existing emissions will increase new emissions. GDP is found to positively affect the CO_2_ emissions, while the renewable energy generation decreases energy-related CO_2_ emissions.

**Fig 3:**
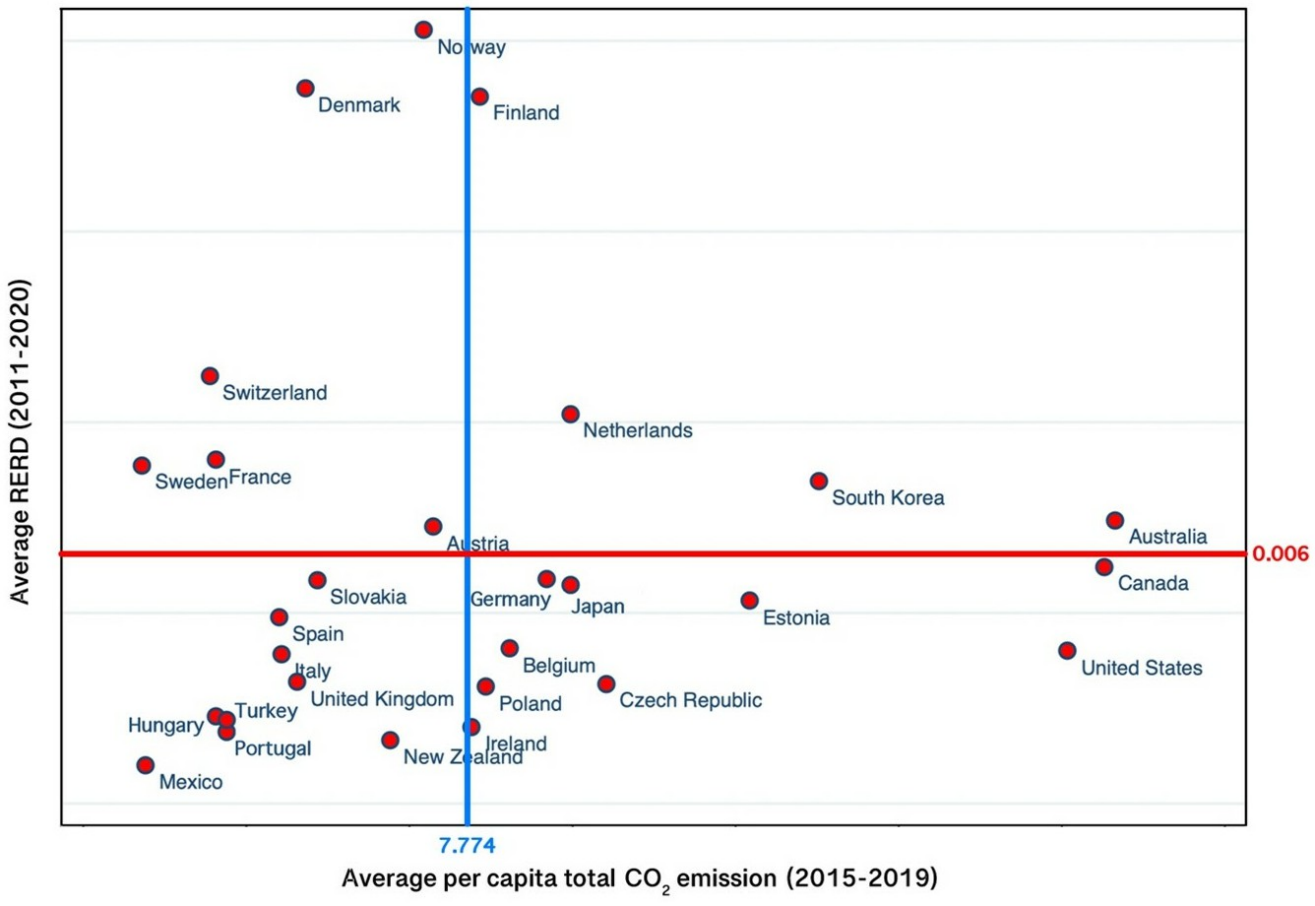
The regional division of selected OECD countries is based on the average values of renewable RD&D percentage of GDP from 2011 to 2020 and the average values of per capita total CO_2_ emissions from 2015 to 2019.

Table 3 displays the estimated results of the two-step system GMM technique across the four categories of the countries depicted in Fig 3. Only in High-RERD and High-Em regions, RERD shows significant effects on CE. A 1% increase in RERD can significantly lead to a 0.033% and 0.05% decrease in CE in High-RERD and High-Em regions, respectively. On the other hand, the effectiveness of *RERD*_*i*,*t*−2_ has not been statistically significant in Low-RERD and Low-Em regions.

The regression results in Table 3 show that renewable energy import has a negative impact on CO_2_ emissions for Low-RERD and Low-Em regions, at 5% and 10% significance levels, respectively. In the contrary, in High-RERD and High-Em regions, no significant effects of REIMP on CE are found.

With regards to the control variables, there is a positive relationship between the economic growth and energy-related CO_2_ emission in all regions. Also, the renewable energy development helps to reduce CO_2_ emissions.

Table 4 also shows the regional reduction effects of renewable energy RD&D and renewable energy import on CO_2_ emissions.

**Table 4:** Regional significant reduction effects of RERD and REIMP on CE.

	High-RERD	Low-RERD	High-Em	Low-Em
*RERD*_*i*,*t*−2_	**↓**		**↓**	
*REIMP*		**↓**		**↓**

The results of robustness test are shown in Table 5. Both the AR(2) and Hansen tests confirm the validity of the model. The robustness examination results further support the results of the main estimation. The consistency in the signs and significance levels of the other variables compared to those in Tables 2 and 3 suggests that the primary estimation findings are robust.

**Table 5:** Results of the robustness test.

Variables	Entire sample	High-RERD	Low-RERD	High-Em	Low-Em
*lnRPAT*_*it*−2_	-0.001	-0.029*	0.010	-0.025**	0.030
	(0.010)	(0.016)	(0.021)	(0.012)	(0.026)
*lnREIMP*	-0.056***	-0.005	-0.112***	-0.061*	-0.097***
	(0.020)	(0.019)	(0.033)	(0.032)	(0.031)
*lnGDP*	0.306***	0.693*	0.623***	0.900***	0.664***
	(0.110)	(0.360)	(0.137)	(0.256)	(0.209)
*lnREDEV*	-0.165**	-0.173*	-0.291***	-0.260***	-0.260**
	(0.072)	(0.093)	(0.064)	(0.066)	(0.095)
*lnEP*	-0.134*	-0.217	-0.418*	-0.154	-0.329
	(0.068)	(0.278)	(0.201)	(0.239)	(0.224)
_*Cons*	-1.208	-4.581**	-1.075	-7.113	-2.355
	(0.990)	(2.154)	(1.192)	(5.321)	(1.704)
*AR*(2)	0.358	0.127	0.017	0.546	0.846
*Hansen test*	0.038	0.998	0.246	1.000	0.257
*Obs*.	211	79	118	85	135

Note: Robust standard errors in paranthesis.

***, ** & * is for 1%, 5% and 10% level of significance.

### 4.2. Discussion

Aligned with OECD [17] that argued certain policies may be suitable and effective in particular contexts, while ineffective or irrelevant in other contexts, The results of this study showed heterogeneous environmental effects of renewable energy RD&D among different regions. There are two potential channels leading to this heterogeneity. The first one is about the amount of the investments in RD&D. Compared with fossil fuels, the renewable energy industry is a capital-intensive industry and needs lots of funds to be invested in R&D [59]. In this regard, Ahmed [14] showed that renewable energy RD&D investment in the US has not gained the required level to affect CO_2_ emissions. Chen [60] also concluded that only when renewable energy investment reaches a certain threshold can renewable energy development be significantly improved. In other words, environmental effectiveness of renewable energy RD&D requires massive public investments to finance huge projects in the sector [61]. Low levels of RD&D expenditures in renewable energy cannot have a significant effect on CO_2_ emissions. This aligns with the finding of this study that argues countries with high RERD expenditures can environmentally benefit from domestic development of renewable energy technologies.

The second channel is about the localization of solutions. Tackling big issues requires the localized solutions. It suggests the huge potential for countries with high levels of carbon emissions to take advantage of domestic renewable energy innovation for climate mitigation. For instance, China, the world’s largest CO2 emitter [62], aiming to move its economy away from coal dependency, has invested heavily in renewable energy [63]. Zhang [64] suggested that the Chinese government should strengthen renewable energy economy technology and self-innovation. In this regard, this study found that countries with massive CO_2_ emissions can benefit from in-house technological innovation in order to achieve the climate goals. This aligns with Cruz-Cázares [65] who showed that innovative firms should rely on the internal development of R&D activities to create barriers to imitation.

In the contrary, the renewable energy RD&D is not a significant determinant of CO_2_ emissions in Low-RERD and Low-Em regions. Countries with low CO_2_ emissions have less need for emission reduction capacities compared to large emitters. Also, it is not worthwhile for low-emitter countries to develop the renewable energy technologies domestically through innovation activities. The results of this study revealed that Low-RERD and Low-Em countries can benefit from technology transfer through the import of renewable energy technologies to reduce the carbon emissions. This is in line with the results of Wagner [66], where he showed that firms acquire R&D when their innovative performance is weak. This result, which indicates the effectiveness of technology transfer in renewable energy, is in line with Erdogan [18], who suggested that the reduction of global CO2 emissions is possible if effective environmental and energy policies are established in international meetings.

Based on the estimated coefficients of the control variables, all of the regions studied in this work can reduce their energy-related CO_2_ emissions by enhancing the level of renewable energy generation. This is consistent with the findings of Yu [67] and Zheng [47]. However, there is a positive relationship between the income level and CO_2_ emission. This result supports the earlier findings of Kahouli [68], Qin [12], Saidi and Omri [69], and Shao [13], and can be explained by the fact that the rise in demand for fossil fuels due to the acceleration of economic activities increases in CO_2_ emissions. Consequently, the environmental quality of countries deteriorates [70].

## 5. Conclusion and policy implications

The current research investigates the impacts of renewable energy RD&D, renewable energy import, economic growth, renewable energy development, and energy productivity on energy-related CO_2_ emissions for OECD member countries over the period from 2011 to 2020.

This study makes five significant contributions: (1) This paper is one of the few studies that investigates the impact of renewable energy RD&D on CO_2_ emissions. This greatly promotes research on the determinants of environmental challenges and effectively complements the related literature. (2) This is the first study to examine the link between renewable energy RD&D and CO_2_ emissions, considering the import of renewable energy technologies as well as economic growth, energy productivity, and renewable energy development. (3) This study applies the CO_2_ emissions from the energy sector. While investigating the environmental impact of renewable energy RD&D, it makes more sense to consider the energy-related CO_2_ emissions. (4) Considering the differences of CO_2_ emissions across various regions, we creatively examine the regional heterogeneous effects of renewable energy RD&D and renewable energy import on CO_2_ emissions. This can provide effective reference for local governments to implement specific and appropriate policies to mitigate the climate change. (5) Due to the delay in the effectiveness of R&D expenditures, we include the lagged term of renewable energy RD&D in the regression model.

The main findings of this study are as follows. (1) For the full panel, CO_2_ emission from energy sector is negatively affected by renewable energy imports and renewable energy development, while GDP has a positive effect on emissions. (2) Only in High-RERD and High-Em regions renewable energy RD&D could decrease CO_2_ emissions, while in Low-RERD and Low-Em regions, increasing the renewable energy import is the effective policy to mitigate emissions. (3) For all four regions, renewable energy development and GDP have decreasing and increasing effects on CO_2_ emissions, respectively.

### 5.1. Policy implications

Considering the remarkable regional differences, the OECD countries should develop specific and appropriate policies and measures compatible with their national circumstances. As discussed, countries are different in CO_2_ emission and renewable energy RD&D. Governments should not necessarily rely on an endogenous development of renewable energy technology to achieve the climate goals, but should formulate a specific strategic plan for their country. Based on the above findings, the following policy recommendations are highlighted.

Firstly, governments that have already invested a lot in renewable energy innovation should allocate still more budgets to renewable energy RD&D in order to reduce carbon emissions. Secondly, it is imperative for countries with relatively high CO_2_ emissions to promote their public investments in renewable energy RD&D in order to localize renewables that reduce emissions.

Thirdly, while renewable energy RD&D doesn’t have a significant effect on CO_2_ emission for Low-RERD and Low-Em countries, these governments could benefit from importing foreign renewable energy technologies to reduce their energy-related CO_2_ emissions. Since the industrial chain of renewable energy in these countries is incomplete, they can benefit from renewable energy technologies by importing different modules of the chain. Our findings suggest policymakers to encourage worldwide cooperation through accelerating renewable energy technology transfer across countries in the form of imports for Low-RERD and Low-Em countries.

Fourthly, in all countries studied in this work, the governments should prioritize ways in which they can effectively promote renewable energy generation. Finally, since the economic growth is associated with the increase in CO_2_ emissions, governments should be conscious of the environmental consequences of the development policies.

### 5.2. Limitations and future research

Nevertheless, due to the lack of data on renewable energy RD&D budgets, this study provides the empirical evidence only for 28 OECD member countries, and is limited to the period 2011–2020. Furthermore, the findings of the present study are based on the public budgets allocated to renewable energy RD&D, while the RD&D investment of the private sector might have a significant impact on CO_2_ emissions too. Therefore, upon availability of data on private renewable energy RD&D budgets, further research would help to understand the environmental impacts of renewable energy technological innovation.

Moreover, we only focus on regional differences and asymmetries in the effectiveness of renewable energy RD&D and renewable energy imports on CO_2_ emissions and do not conduct in-depth research on how other independent variables affect CO_2_ emissions. Therefore, it would be interesting to add other factors to our econometric model that may potentially play a significant role in the CO_2_ emissions from energy.

## Supporting information

S1 Appendix (DOCX)

S1 Dataset (XLSX)
