# Supplementary material for: Innovation or acquisition? Emissions mitigation strategies and the role of renewable technologies
Source: PLoS One. 2024 Dec 31;19(12):e0316020. doi: 10.1371/journal.pone.0316020 (PMC11687885; doi:10.1371/journal.pone.0316020)
Supplement: S1 Appendix — (DOCX) [file pone.0316020.s001.docx]

# **Appendix**

Table A1. Correlation matrix.

|  | CE | RERD | REIMP | GDP | REDEV | EP |
| --- | --- | --- | --- | --- | --- | --- |
| CE | 1.00 |  |  |  |  |  |
| RERD | -0.01 | 1.00 |  |  |  |  |
| REIMP | 0.07 | 0.28 | 1.00 |  |  |  |
| GDP | 0.08 | 0.29 | 0.14 | 1.00 |  |  |
| REDEV | -0.30 | 0.29 | -0.05 | 0.42 | 1.00 |  |
| EP | -0.52 | -0.02 | -0.05 | 0.49 | 0.20 | 1.00 |

Table A2. Panel unit root tests.

| Variables | ADF (1 lag) | | ADF (2 lags) | |
| --- | --- | --- | --- | --- |
|  | Level | First Difference | Level | First Difference |
| $lnCE$ | -2.73 | -0.70 | 3.04*** | 4.42*** |
| $lnRERD$ | 0.41 | 16.48*** | -2.37 | 0.13 |
| $lnREIMP$ | 9.21*** | 14.54*** | 6.41*** | 6.47*** |
| $lnGDP$ | 3.84*** | -3.07 | 5.89*** | 3.88*** |
| $lnREDEV$ | -0.40 | 6.17*** | -1.12 | 6.87*** |
| $lnEP$ | -1.41 | 12.41*** | 3.33*** | 5.76*** |

Note: ***, ** & * is for 1%, 5% and 10% level of significance.

Table A2 shows the estimated results of the ADF unit root test. REIMP and GDP are stationary at level; and RERD, REDEV, and EP are stationary at first difference; with one lag used to remove higher-order autoregressive components of the series. After adding one other lag, CE also passes the unit root test.

Table A3. Cointegration tests.

| Variable | Kao (ADF statistics) | Westerlund | |
| --- | --- | --- | --- |
|  |  | No Trend, No demean | Trend, demean |
| $lnRERD$ | 3.23*** | 0.33 | 1.65** |
| $lnREIMP$ | 1.66** | 3.89*** | 1.42* |
| $lnGDP$ | 1.98** | 0.66 | 1.49* |
| $lnREDEV$ | 1.66** | -1.87** | 2.70*** |
| $lnEP$ | -1.51* | -3.11*** | 3.06*** |

Note: ***, ** & * is for 1%, 5% and 10% level of significance.

The estimated results for cointegration via employing Kao and Westerlund tests are provided in Table A**3**. The Kao cointegration test rejects null hypothesis that no cointegration exists among the variables. Instead, the alternative hypothesis of the presence of the long-run cointegration could be accepted, which indicates the long-run association among the variables. Similarly, the Westerlund cointegration test, subtracting cross-sectional means, discloses that all the variables are cointegrated. The results are statistically significant at 1%, 5%, and 10% levels.

Table A4. The countries of the four regions.

| Region | Countries |
| --- | --- |
| High-RERD | Australia, Austria, Denmark, Finland, France, Korea, Netherlands, Norway, Sweden, Switzerland |
| Low-RERD | Belgium, Canada, Czech Republic, Estonia, Germany, Hungary, Ireland, Italy, Japan, Mexico, New Zealand, Poland, Portugal, Slovak Republic, Spain, Turkey, United Kingdom, United States |
| High-Em | Australia, Belgium, Canada, Czech Republic, Estonia, Finland, Germany, Ireland, Japan, Netherlands, Poland, South Korea, United States |
| Low-Em | Austria, Denmark, France, Hungary, Italy, Mexico, New Zealand, Norway, Portugal, Slovakia, Spain, Sweden, Switzerland, Turkey, United Kingdom |
